# Infliximab versus ciclosporin in steroid resistant acute severe ulcerative colitis: a model-based cost-utility analysis of data from CONSTRUCT pragmatic trial

**DOI:** 10.1186/s12913-023-09233-w

**Published:** 2023-03-08

**Authors:** Mohammed Fasihul Alam, Mirella Longo, David Cohen, Sam Groves, Laith Alrubaiy, Hayley A. Hutchings, Alan Watkins, Shaji Sebastain, John G. Williams

**Affiliations:** 1 Department of Public Health, College of Health Sciences, QU-Health, Qatar University, PO Box 2713, Doha, Qatar; 2 Marie Curie Palliative Care Research Centre, Cardiff University, 8th Floor Neuadd Meirionnydd, Heath Park Way, Cardiff, CF14 4YS UK; 3 Faculty of Health, Sport and Professional Practice, University of South Wales, Lower Glyntaff, Pontypridd, CF37 1DL UK; 4 Swansea Centre for Health Economics, College of Health and Human Sciences, Swansea University, Singleton Park, Swansea, SA2 8PP UK; 5 Swansea University Medical School, Swansea University, Singleton Park, Swansea, SA2 8PP UK; 6 IBD Unit, Hull University Teaching Hospitals NHS Trust, Hull, UK

**Keywords:** Ulcerative colitis, Infliximab, Ciclosporin, Cost-effectiveness, Decision tree, Markov model

## Abstract

**Background:**

There is limited evidence in the literature on the long-term effectiveness and cost-effectiveness of treatments for Acute Severe Ulcerative Colitis (ASUC). The study aimed to perform decision analytic model-based long-term cost-utility analysis (CUA) of infliximab versus ciclosporin for steroid-resistant ASUC investigated in CONSTRUCT pragmatic trial.

**Methods:**

A decision tree (DT) model was developed using two-year health effect, resource use and costs data from CONSTRUCT trial to estimate relative cost-effectiveness of two competing drugs from the United Kingdom (UK) National Health Services (NHS) perspective. Using short-term trial data, a Markov model (MM) was then developed and evaluated over further 18 years. Both DT and MM were combined to investigate cost-effectiveness of infliximab versus ciclosporin for ASUC patients over 20-year time horizon, with a rigorous multiple deterministic and probabilistic sensitivity analyses to address uncertainty in results.

**Results:**

The decision tree mirrored trial-based results. Beyond 2-year trial follow-up, Markov model predicted a decrease in colectomy rate, but it remained slightly higher for ciclosporin. NHS costs and quality adjusted life years (QALYs) over base-case 20 year time horizon were £26,793 and 9.816 for ciclosporin and £34,185 and 9.106 for infliximab, suggesting ciclosporin dominates infliximab. Ciclosporin had 95% probability of being cost-effective at a willingness-to-pay (WTP) threshold value up to £20,000.

**Conclusion:**

Using data from a pragmatic RCT, the cost-effectiveness models produced incremental net health benefit in favour of ciclosporin relative to infliximab. Results from long-term modelling indicated that ciclosporin remains dominant compared with infliximab for the treatment of NHS ASUC patients, however, these need to be interpreted cautiously.

**Trial registration:**

CONSTRUCT Trial registration number ISRCTN22663589; EudraCT number: 2008-

001968-36 (Date 27/08/2008).

**Supplementary Information:**

The online version contains supplementary material available at 10.1186/s12913-023-09233-w.

## Introduction

Ulcerative colitis (UC) is a chronic inflammatory disorder of the colon, characterised by mucosal ulceration, rectal bleeding, diarrhoea and abdominal pain [1] all of which have considerable impact on patients’ quality of life. UC affects about 150,000 people in the United Kingdom (UK) and 2 million people in Europe [2, 3]. Approximately one-quarter of these patients are affected by acute severe ulcerative colitis (ASUC), and require hospital admissions for treatment with intravenous steroids [4]. However, about 30–40% of these patients are steroid resistant [5, 6]. In the absence of other treatments, previously colectomy was the only available option for treating these patients [6]. Although mortality following emergency colectomy has fallen over time, 10% of patients die within 3 months of surgery [7]. The use of intravenous or oral ciclosporin, a calcineurin inhibitor that selectively inhibits T-cell function, and infliximab, a monoclonal antibody that targets tumour necrosis factor α, has offered hope for avoiding colectomy in steroid-resistant UC [8–11].

A number of previous studies have confirmed the efficacy of infliximab and ciclosporion in the treatment of patients with moderate or severe steroid-resistant UC [12–16]. As a rescue therapy, both infliximab and ciclosporion have appeared to be equally effective in preventing colectomy in both short- and long-term follow-up [16]. Although colectomy rate was higher for ciclosporin at 3 m and 12 m follow-up, at 36 m follow-up it was lower for ciclosporin compared to infliximab patients.. A recent systematic review also concluded that both drugs are equally effective as rescue therapy to reduce colectomy rates in the short-term, however, in the long-term infliximab is better than ciclosporin in terms of avoiding colectomy [17].

A European randomised controlled trial (CYSIF) found no statistically significant difference between infliximab and ciclosporin in clinical effectiveness at 12 months but the study did not report on cost-effectiveness of the treatments [18]. The evidence on the long-term clinical- and cost-effectiveness of these drugs is limited in the literature. Two studies have investigated relative cost-effectiveness using modelling methods but have not used data from head to head randomised trials, systematic reviews or meta-analyses [19, 20].

CONSTRUCT was an open-label, multicentre, parallel-group, pragmatic randomised clinical trial (RCT) with 3-years follow up (FU). A total of 270 adults admitted with acute severe ulcerative colitis who failed to respond to 2–5 days intravenous hydrocortisone were recruited from 52 district general and teaching hospitals across England, Scotland and Wales [21]. Patients randomised to infliximab received Remicade® (5 mg/kg intravenous infusion) and those randomised to ciclosporin received Sandimmun® (by continuous infusion of 2 mg/kg/day). Full details of these trial treatments are reported elsewhere [22, 23]. The study has shown that ciclosporin patients have produced non-significant higher quality of life values than infliximab patients at different follow-up time points and that the healthcare services cost is significantly higher for infliximab, mainly due to higher acquisition cost [22]. That analysis only estimated the relative cost-effectiveness of two drugs over the trial follow up period and did not provide estimates of long-term cost-effectiveness [24].

At the time of the CONSTRUCT trial infliximab was still on patent as Remicade®. When the patent expired in 2015 biosimilars such as Inflectra® and Remsima® were launched in the UK with an NHS list price of £377.66 per 100 mg vial, which is 10% lower than the list price of Remicade® (£419.62 per 100 mg vial) [25], Inflectra® has shown similar efficacy and safety as Remicade® in adult severe UC patients [26]. Although British National Formulary (BNF) list price of these biosimilars is approximately 10% lower than the Remicade’s price, local hospitals’ current drug acquisition costs for infliximab biosimilars might be much lower. It is therefore important to investigate the impact of the price-reduction of infliximab on the relative cost-effectiveness of two alternative drugs for treating patients with acute severe UC.

In this study, we have developed and validated decision analytical models that allowed extrapolation of health effect and resource use implications of competing treatments beyond trial follow up period and estimation of long-term cost-effectiveness of infliximab versus ciclosporin for steroid resistant ASUC patients.

## Methods

We used data from 270 ASUC patients in the CONSTRUCT pragmatic study to develop a decision tree and a Markov state-transition model. The study considered the UK National Health Services (NHS) perspective and adopted a cost-utility analysis (CUA) approach. NHS resource usage, including intervention drugs, and their costs captured in the trial were modeled and extrapolated over the long-term study duration. Patients recruited in the trial were 18 years or older who failed to respond to 2–5 days of intravenous hydrocortisone with continuing severe disease. Mean (SD) age of patients at randomisation was 39.3 (15.5) in Infliximab and 39.8 (15.0) in Ciclosporin group, respectively. Patients randomized to infliximab were given 5 mg/kg by intravenous infusion over 2 h at baseline, and again at 2 weeks and 6 weeks after the first infusion, in line with local hospital guidelines. Ciclosporin patients received Sandimmun by continuous infusion continued for up to 7 days if successful, and then switched to twice-daily oral doses. After 12 weeks, all treatment was at the discretion of patient’s physician in the respective hospital. Most of the ciclosporin patients completed their treatment within first 12 weeks but for a number of infliximab patients drug costs continued during the follow up.

The study followed the UK NICE’s guide to methods of technology appraisal for economic evaluation and base in British National Formulary (BNF) pricing for valuing both treatment drugs used within the NHS. Infliximab biosimilars are likely to push down drug acquisition costs for local hospitals through commercial negotiations in confidential, however, these costs might not be true representative of actual drug costs of the manufacturer, hence, not been used for economic costing in the study. However, the change in the price for infliximab biosimilars and its potential impact on cost-effectiveness results have been investigated via deterministic sensitivity analyses. Infliximab was priced according to the BNF listed price which has been unchanged since 2012–2013.

A CUA is adopted to evaluate the cost-effectiveness of alternative treatments in ASUC patients, and to provide evidence for policy makers on reimbursement decisions, which uses Quality Adjusted Life Year (QALY) as a health outcome. CUA evaluates the cost-effectiveness of infliximab versus ciclosporin in terms of costs and QALY implications. An incremental cost-effectiveness ratio (ICER) is estimated (if an intervention appears to be more effective and more costly than a comparator) and compared with the UK National Institute for Health and Care Excellence (NICE) recommended willingness-to-pay threshold value, £20,000 per QALY gain. Following NICE recommendations a discounting rate of 3.5% per annum was applied to both costs and QALYs occurring beyond 12 months from two alternative treatments.

### Short-term decision tree (DT) model

Given the acute phase of the disease, a decision analytical (decision tree) model was developed and adapted from Punekar and Hawkins [20] to simulate the progression of a cohort of steroid-resistant severe UC patients receiving infliximab or ciclosporin. The associated costs and outcomes were tracked over 2-year time horizon to capture medium-term colectomy risks as observed in clinical studies. Treatment outcomes in the DT model were characterised into three time periods: 0–3 months, 4–12 months and 13–24 months. The model was built using Microsoft Excel, where treatment options and patient pathways are depicted in Appendix 1.

Following treatments with infliximab or ciclosporin, ASUC patients either achieved remission, or failed treatment and underwent colectomy. It was assumed that if treatments failed, patients underwent a colectomy [19, 20].

### Model assumptions

1. All patients who achieved remission would maintain it during the first period (0–3 months).
2. During the second period (4–12 months), patients who achieved the initial remission would either maintain it for the rest of 9 months or if lost responses would undergo colectomy. For patients had colectomies in the second period the model assumed that colectomies occurred mid-period i.e. at 8 months.
3. During the third period (13–24 months), patients who achieved the initial remission would either maintain it for the rest of 12 months or if lost responses would undergo colectomy. Patients who had colectomies in the third period the model assumed that colectomies occurred mid-period i.e. at 18.5 months.
4. After surgery, patients either achieved remission or experienced post-surgery complications immediately after the surgery in the same period. Those treated for post-surgery complications, achieved post-surgery remission in the next period and maintained it for the rest of the analysis.

### Long-term cost-effectiveness using a Markov model (MM)

A Markov model (Appendix 2) was constructed to estimate the long-term cost-effectiveness of infliximab versus ciclosporin beyond the trial follow-up period. The cohort of UC patients entered into the Markov model after completing first 2 years in DT. The model had a time cycle of one year, and forecasted the impact of long-term probability of colectomy on costs and QALYs over 18-year time horizon. The model assumed that those patients who attained remission from any of two trial drugs could remain in the remission state for the whole analysis period or lose response and undergo for colectomy, and after surgery they achieve surgical remission or can die. It was assumed that post-colectomy complications occur immediately after the surgery in the same cycle as surgery, and after treatment for complications they recover in the following cycle, achieve surgical remissions or can die.

A total of 56% UC patients in the trial were without a colectomy at the last follow-up, and the number (%) of colectomies in trial patients at different follow-up time points is shown in Appendix 3. We estimated a Weibull regression [27] to estimate time-to-colectomy data over 2 years follow-up period (only one patient had a colectomy after 2 years FU), with age-at-randomisation and weight as covariates. The Weibull scale (lambda) and shape (gamma) parameter estimates were linked to extrapolate time-dependent transition probabilities of colectomy over 18-year time horizon [28]. For comparisons, we also estimated a number of other regressions e.g. Gompertz, exponential, log-logistic to estimate time-to-colectomy data, and compared performance of these models using AIC (Akaike Information Criterion) and BIC (Bayesian Information Criterion) values.

There was no reported mortality in one of the treatment arms of the CONSTRUCT trial. The study accounted for the impact of within-trial mortality in the cost-effectiveness DT model. For long-term modelling in the Markov model, the study considered 3-year mortality data from a published observational study conducted in England for ulcerative colitis and Crohn’s patients during 1998–2003 [29]. Following clinical experts’ opinion, our study only considered mortality for ‘emergency colectomy’ and ‘no colectomy’, excluded mortality for ‘elective colectomy’ patients from the observational study. The 3-year mortality rate was then converted into a yearly rate to be used in the Markov model, following appropriate steps mentioned in Briggs et al. [28]. Apart from the health state mortality rates, other parameter values used to run the Markov model are based on data from the CONSTRUC trial.

### Utilities

The utility values (health related quality of life (HRQoL) weights) used in DT pathways and Markov health states are from the CONSTRUCT trial data (for Markov model, these values are presented in Appendix 4). Utility values of trial patients experienced with different health ‘states’ were plugged into the Markov model. The HRQoL values in the original trial are based on EuroQoL-5 dimension-3 level (EQ-5D-3L) questionnaire to estimate trial patients’ quality of life score captured at baseline, 3 m, 6 m, 12 m and then at every 6 m intervals from 1 to 3 years follow-up period. EQ-5D-3L assesses HRQoL on five dimensions – mobility, self-care, usual activities, pain or discomfort, and anxiety or depression – using three levels for each dimension [30]. The study used a UK tariff to convert these into a single utility score. In the trial, infliximab patients started with non-significant higher utility values than ciclosporin at baseline. However, from 6 m onward at each follow-up period up to 30 m, ciclosporin patients showed higher utility values than infliximab and the difference was not statistically significant. Details of the utility data captured in the CONSTRUCT trial were reported elsewhere [22].

### Costs

The decision models are based on healthcare services use and costs captured in the CONSTRUCT trial. NHS resource use were collected from case report forms (CRFs) and participant follow-up questionnaires (PFQs) completed at each follow-up time points, supplemented by post-colectomy questionnaire, SAE forms and relevant data provided by participating sites. The costs of all healthcare services use were estimated using standard NHS Pay and Price index. These included trial drugs, their preparation and administration costs, NHS contacts, consultant (clinic visit, telephone call, dietician), primary care general practitioner (at practice, home visit, telephone call), health visitor, nurse specialist, tests and investigations, hospitalizations (with and without surgery), readmissions, etc. Details on NHS resource uses, how these were captured and their costs (in 2012–13 prices in Great Britain Pound (GBP) sterling) were reported in the CONSTRUCT main report [22] hence not repeated in the manuscript, however, in this study an adjustment was made by using consumer price index (CPI) to inflate these costs to the year 2019 [31]. The unit costs of the two trial drugs are given in Appendix 5.

### Sensitivity analysis (SA)

The study employed multiple deterministic and probabilistic sensitivity analyses (PSA) to address (parameter) uncertainty with cost-effectiveness results.

#### Deterministic SA – reduction in Infliximab biosimilars’ price

Infliximab biosimilar (Inflectra® and Remsima®) have been indicated for a number of conditions within the NHS, and evidence shows these biosimilars appear to be equally effective as infliximab in patients with UC. The price of these biosimilars has been decreasing, and in the BNF currently it is 10% less than the price of Remicade. The study also considers a major reduction in biosimilar price such as 50%, 70% and 80% of the Remicade’s list price.

#### Deterministic SA – changes in health utilities

Given uncertainties in relative effectiveness of two competing treatment options from the literature, we also investigate the impact of ± 10% change in the base-case utilities.

#### Deterministic SA – 10 years of evaluation period

The SA shows whether the cost-effectiveness results are sensitive to a shorter time horizon of 10 years, as compared with the base-case 20 years time horizon.

#### Deterministic SA – fixed transition probability of colectomy

We also investigated the impact of a fixed-transition probability of colectomy in Markov model. In the trial, colectomy rates were very low in both groups beyond 2 years follow-up. So, we considered colectomies that occurred during 4–24 months period, converted these rates into a yearly probability following steps mentioned in Briggs et al. [28] and applied this as a fixed-transition probability of colectomy in the Markov model. A separate fixed transition probability of colectomy was considered for both infliximab and ciclosporin treatment groups.

#### Probabilistic sensitivity analysis (PSA)

Probabilistic sensitivity analysis (PSA) was conducted by considering an appropriate probability distribution for Markov model parameters that included utility values, Weibull parameters, mortality rates and costs of resource use with health ‘states’. For example, a beta probability distribution was considered for Weibull scale (lambda) and shape (gamma) parameters and utilities for both infliximab and ciclosporin groups, while cost was varied using a Gamma distribution. The mean costs and QALYs are subject to sampling error. The effect of parameter uncertainty on cost-effectiveness results was addressed by employing a Monte Carlo (MC) simulation to generate 10,000 input configurations for model parameters and then the Markov model was run with each parameter set. Results from the PSA were presented via a CE plane and CEAC curves.

Model-based cost-effectiveness results in the study were produced based on Consolidated Health Economic Evaluation Reporting Standards (CHEERS) checklist [32].

### Patient and public involvement

ASUC patients and the public were not involved in the design, conduct or reporting of this research.

## Results

### Decision tree (DT)

Cost-effectiveness results from a short-term decision tree model are presented in Table 1. The model produced similar results to that of within-trial 24 month follow-up data, representing an internal validity of model results. The difference in QALYs (ciclosporin-infliximab) is 0.035 (Table 1), which is in favour of ciclosporin and almost in line with what observed in within-trial analyses of 24 months follow-up data. Ciclosporin appeared to be both more effective and less costly than infliximab. The estimated NHS cost produced from the model was £11,705 and £18,608 for ciclosporin and infliximab group, respectively.

**Table 1:** Base-case cost-effectiveness results from a decision tree and Markov model

	**Infliximab**		**Ciclosporin**		**Difference (ciclos – inflx)**		**Cost-effectiveness**
	**Costs (£)**	**QALYs**	**Costs (£)**	**QALYs**	**Costs (£)**	**QALYs**	
Decision tree (2-year)	18,608	1.561	11,705	1.596	-6,902	0.035	Ciclosporin dominates
Markov model (beyond 2-year trial FU and over 18 years)	15,748	7.613	15,103	8.219	-645	0.606	Ciclosporin dominates
Base-case (20-yr time horizon); DT + MM	34,185	9.106	26,793	9.816	-7,392	0.710	Ciclosporin dominates

*FU* Follow Up, *DT* Decision Tree, *MM* Markov Model

### Long-term cost-effectiveness from the MM

We have produced Kaplan–Meier (KM) colectomy-free survival curves for infliximab and ciclosporin over the CONSTRUCT trial follow-up period, which show that there is a separation in the curves, however, this was not statistically significant (Log rank test, *p* = 0.24) (Appendix 6).

Table 2 presents results from the estimated Weibull hazard function for both infliximab and ciclospsorin. Although we tried a number of other parametric functions, e.g. log-logistic, Gompertz and exponential to estimate time-to-colectomy data, a Weibull probability distribution produced a better fit of the data based on AIC and BIC values. None of the covariates appeared to be significant for infliximab, however, they were significant for ciclosporin. The hazard function is extrapolated beyond the range of the trial follow-up period for up to 18 years. The purpose of this function is to predict the risk of colectomy for each cycle in the cost-effectiveness model.

**Table 2:** Results from the Weibull regression function to estimate time-to-colectomy data for infliximab and ciclosporin from the CONSTRUCT trial

	**Infliximab**			**Ciclosporin**		
	**Hazard ratio**	**SE**	***p*-value**	**Hazard ratio**	**SE**	***p*-value**
Age-at-randomisation	1.009	0.008	0.256	1.022	0.009	0.009
Weight	0.989	0.010	0.305	0.976	0.010	0.013
Gamma (shape parameter)	0.393	0.047		0.450	0.047	
Lambda (scale parameter)	0.111	0.097	0.012	0.186	0.144	0.030

*SE* Standard Error

A significant Gamma value in Table 2 indicates that there is a time-dependent transition probability of colectomy for a yearly cycle length, which was decreasing over time. Appendix 7 presents yearly transition probabilities of colectomy (%), based on the Weibull model, in patients with infliximab and ciclosporin. The model predicted a decreasing colectomy rate for both groups, however, a higher colectomy rate remained for ciclosporin.

Table 1 presents the base-case cost-effectiveness results over 20 years time horizon by combining a short-term DT and long-term Markov model, along with deterministic SA results presented in Table 3. Base-case cost-effectiveness results suggest that ciclosporin was more effective and less costly than infliximab, that is, ciclosporin dominates infliximab.

**Table 3:** Cost-effectiveness results from one-way deterministic sensitivity analyses

Scenario	Infliximab		Ciclosporin		Incremental QALY (Ciclos-Inflx)	Incremental Cost (Ciclos-Inflx)	ICER/Cost-effectiveness
	**Costs (£)**	**QALYs**	**Costs (£)**	**QALYs**			
10% reduction in inflx. price	33,331	9.106	26,793	9.816	0.710	-6,538	Ciclsoporin dominates
20% reduction in inflx. price	32,477	9.106	26,793	9.816	0.710	-5,684	Ciclsoporin dominates
50% reduction in inflx. price	29,914	9.106	26,793	9.816	0.710	-3,121	Ciclsoporin dominates
70% reduction in inflx. price	28,206	9.106	26,793	9.816	0.710	-1,413	Ciclsoporin dominates
80% reduction in inflx. price	27,352	9.106	26,793	9.816	0.710	-559	Ciclsoporin dominates
10% reduction in ciclos. utility	34,185	9.106	26,793	9.708	0.602	-7,392	Ciclosporin dominates
10% increase in ciclos. utility	34,185	9.106	26,793	9.913	0.807	-7,392	Ciclsoporin dominates
10% reduction in inflx. utility	34,185	8.951	26,793	9.816	0.865	-7,392	Ciclsoporin dominates
10% increase in inflx. utility	34,185	9.262	26,793	9.816	0.554	-7,392	Ciclosporin dominates
Fixed transition probability (TP)	44,829	9.283	41,005	9.721	0.438	-3,824	Ciclsoporin dominates
10-year time horizon (time dependent TP)	28,163	6.315	20,376	6.713	0.398	-7,787	Ciclsoporin dominates
10-year time horizon (Fixed TP)	31,326	6.198	24,963	6.475	0.277	-6,363	Ciclsoporin dominates
Beyond 2-yr trial follow-up	15,748	7.613	15,103	8.219	0.606	-645	Ciclsoporin dominates

*DT* Decision Tree, *TP* Transition Probability

### Deterministic and probabilistic sensitivity analyses

The models predicted the most favourable results for ciclosporin (more effective and less costly) in all twelve different scenarios considered for one-way deterministic sensitivity analyses (Table 3). One deterministic SA included fixed-transition probabilities from remission to surgery health state by converting 4–24 months colectomy rates from the trial into yearly fixed-transition probability of colectomy of 0.07 for infliximab and 0.10 for ciclosporin. Results from all twelve different one-way SA scenarios produced cost-effectiveness results showing ciclosporin dominated infliximab.

**Fig. 1:**
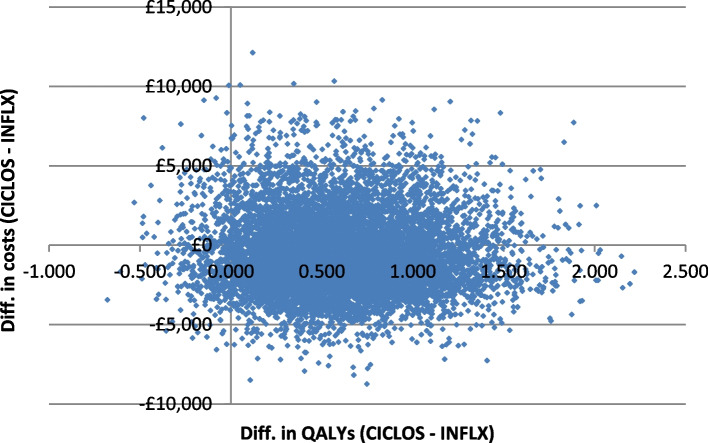
Cost-effectiveness plane showing parameter uncertainty in costs and QALYs from the Markov model via 10,000 Monte Carlo simulations of the input configurations. X-axis and Y-axis were showing both difference in QALYs and costs, respectively, between infliximab and ciclosporin

**Fig. 2:**
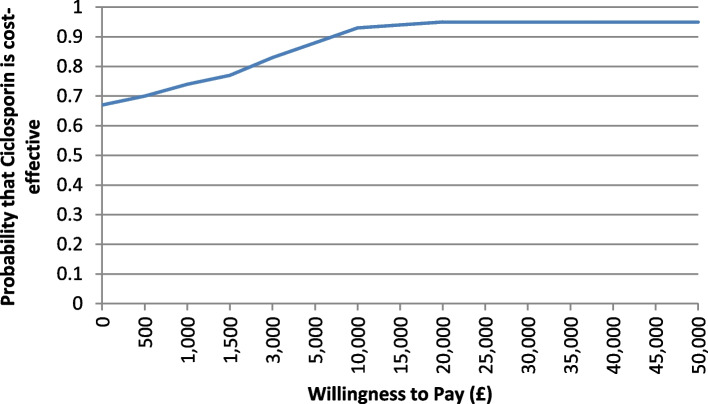
CEAC curve showing the probability that ciclosporin is cost-effective against a range of WTP threshold values

Figures 1 and 2 represent a CE plane and CEAC curves which are produced to show cost-effectiveness results from a probabilistic sensitivity analysis. Compared to infliximab, there is a 95% probability that ciclosporin will be cost-effective at a WTP of up to £20,000 per QALY gain (Fig. 2).

## Discussion

We developed and used decision analytical models to investigate long-term cost-effectiveness of infliximab versus ciclosporin in the management of steroid-resistant ASUC patients. The study used data from the RCT to extrapolate colectomy beyond the trial follow up and to estimate its health effect (QALYs) and resource use implications over 20 years time horizon. Results from both decision tree and Markov models suggest that ciclosporin is more effective in terms of QALY gain and less costly than infliximab. Although the difference in QALYs between two groups was not statistically significant in within-trial analyses, infliximab appeared to be significantly more costly in terms of the NHS healthcare costs [22]. This suggests ciclosporin dominates infliximab in the management of severe, acute UC patients with some associated uncertainties. The probabilistic sensitivity analyses from long-term cost-effectiveness model suggest that ciclosporin had 95% probability of being cost-effective compared to infliximab at a willingness-to-pay (WTP) value of £20,000 per QALY gain, currently used by NICE. This probability was 74% in within-trial cost-effectiveness analysis [22]

An evidence review committee of NICE expressed concerns over the uncertainty in clinical effectiveness of the treatments, e.g. colectomy rates, due to very small number of RCTs, which themselves were small, and to criticism of the use of evidence from mixed treatment comparisons [33]. Our study provided the opportunity to access trial data from a sufficiently powered head-to-head comparison and facilitate both short-term ‘within-trial’ and long-term model-based cost-effectiveness analyses of two competing treatments for UC patients.

The short-term decision tree model with 2-year time horizon has produced similar cost-effectiveness results to those from a 24-month ‘within-trial’ analysis, reflecting an internal validity of the model and its results [22]. Both differences in QALYs and costs between two treatments were in the same direction, although the magnitude of the difference in costs was found to be relative higher from the decision tree model.

The model predicted the risk of colectomy among UC patients beyond 2-year follow up period, hence, its long-term implications on both QALYs and costs. Given a very few patients had colectomy after 24 months, we based our time-to-colectomy survival analysis using 24 months follow-up data so that a better prediction in the right-hand tail of survival curves would be achieved. However, this remained as one of the study limitations. An RCT with relatively a higher sample size could potentially reduce this limitation. Another limitation of the study is—not considering a societal cost perspective, resulting in a potentially conservative approach. Indirect costs such as absence from work due to the disease were not considered in the study, which fall outside the healthcare sectors. However, indirect costs may exceed direct costs since IBD is generally diagnosed in early adulthood or middle-age with relatively more productivity among this population [34]. But competing treatments for AUSC patients may generate more surgeries and hospitalisations, means that it may lead to more production losses. Given a higher colectomy rate was observed in ciclosporin patients, this group then might have experienced greater average cost than infliximab under a societal perspective.

Arguably hospitals’ drug acquisition costs for infliximab might be significantly lower than the BNF listed price. This is due to fact that a drug company may negotiate a price below its cost in order to get more future business from hospitals in the region, nevertheless, the negotiated price would not represent its value. The principle in economic evaluation is to put a money value on a healthcare resource not to measure the cash paid for it. However, the study provides the potential changes in infliximab biosimilars’ price and its impact on cost-effectiveness results through robust sensitivity analyses, which favours ciclosporin as a cost-effective treatment option.

Beyond 2-year follow-up, ciclosporin remained more effective and less costly than infliximab over 18-year time horizon, irrespective of varied risk of colectomy between groups in the model. Although the model projected slightly higher colectomy rates for ciclosporin patients, in line with higher within-trial colectomies for this group, the estimated ‘overall’ higher cost for infliximab group is due to its associated higher average cost of ‘remission from medicine’ health state that is propagated through the Markov model. Another likely explanation of higher average costs associated with infliximab group is that treatment with ciclosporin tended to be stopped by 12 weeks, whereas those on infliximab tended to continue treatment for longer. This was at the discretion of the attending physician and reflected the pragmatic nature of the trial [22]. The cessation of ciclosporin earlier than infliximab may explain why the colectomy rate was higher in this group, although quality of life after colectomy was generally better [35]. As found in a number of studies, surgery did not adversely affect the QoL of ASUC patients [36, 37]. Moreover, patients in the infliximab group experienced higher number of serious adverse reactions (SARs) than ciclosporin group. For example, there were eight infection related SARs in infliximab versus one in ciclosporin group, respectively [23]. The other major clinical trial on ASUC also reported higher number of serious adverse events (SAEs) in infliximab, compared to ciclosporin patients [18].

In our cost-effectiveness model, mortality from different health states is considered from a published study which showed small but higher mortality for ‘no colectomy’ than ‘emergency colectomy’ UC patients [29]. As outlined in methods, the study assumed mortality from ‘no colectomy’ means mortality from ‘remission’ following alternative treatments. It did not consider mortality from ‘elective’ colectomy which is not usually offered when patients are in remission and stable, unless investigations had detected unstable mucosal cells indicating a risk of cancer. Also, the mortality among patients who did not have a colectomy following unscheduled/emergency admission was as high as those who had a colectomy because they were either too ill for surgery or this was contraindicated because of other serious health problems [29].

By combining the decision tree with the long-term Markov model, base-case cost-effectiveness results suggested that ciclosporin dominates infliximab. The base-case cost effectiveness results were remained robust to changes in different factors, which can be seen from deterministic one-way sensitivity analyses. Overall, this confirms that ciclosporin appears to be more cost-effective treatment for ASUC patients in the UK, however, the difference in costs between two treatment options is apparent globally [38, 39].

The study is based on utility values from 3-year follow up data collected in the CONSTRUCT trial, which showed an utility decrement among colectomy patients in the short term. Given higher colectomies in ciclosporin patients, this in fact disfavours ciclosporin, compared to infliximab, in terms of utility gain. However, in the long term surgery did not result in decrement in quality of life for UC patients [35, 36], may be due to the fact that it might lead to remission after surgery. From the trial-based economic evaluation, the study team showed non-significant differences in QALY but it was favouring ciclosporin. This may raise the question whether adding a non-significant QALY gain for ciclosporin over 20 years is justifiable or not. We argue that, the study attempts to estimate the cost and effect differences and to quantify the likelihood of an intervention being cost-effective, while considering a fundamental analytical issue of an economic evaluation- joint uncertainty in both costs and effects [40]. As analysts, it is not our role to determine whether a difference in costs or QALYs is ‘acceptable’ or not.

Our results differ from those found in a model-based study by Punekar and Hawkins [20] that produced infliximab to be more effective and more costly than ciclosporin, with an ICER £19,545 which is viewed as cost-effective when compared with current NHS willingness-to-pay threshold. This was followed by another model-based study in the Netherlands which produced an ICER of €24,277 [38]. However, some of the assumptions made in those studies may not entirely reflect current clinical practice. Both studies considered that infliximab patients receive three infusions as per protocol, whereas in the CONSTRUCT trial treatment with infliximab was at the discretion of the respective consultant who provided more than three infusions to 25% of the infliximab patients and up to 13 infusions in one case. Also, the cost-effectiveness results from above model based studies appeared to be sensitive to patients’ body weight considered, e.g. 80 kg and 70 kg per patient in the UK and Netherlands, respectively. In contrast, treatment of patients in both arms of the CONSTRUCT trial was based on their actual average weights.

Standard parametric models are generally fitted to time-to-event data from trials for future projection of health events such as colectomy. However, there are inherent and unavoidable limitations in estimating parametric functions as not enough data available from short follow-ups to compare long-term outcomes from the model [41]. These functions may differ in underlying assumptions, e.g. hazards of the event might be time-dependent (e.g. Weibull, Gompertz) or time-independent (exponential) [42]. One could also argue that different parametric models should be estimated for time to colectomy data and compared One of the study limitations is that, it considered only a Weibull distribution which appeared to provide better goodness-of-fit for time to colectomy data from the CONSTRUCT trial. Curve fitting and statistical methods used to assess relative goodness-of-fit of event data may not be necessarily producing clinically acceptable results when projected into the future, since many aspects of patient care settings are subject to ongoing changes [43]. On the contrary, standard mathematical models are generally continuous smooth functions without an abrupt alteration in the trend [43].

It is evident from observational studies that the cumulative rate of colectomies increases over time with both infliximab and ciclosporin [44–47]. Hence, the CONSTRUCT investigation team has been following up the trial and cohort participants of the study for 10 years from recruitment using routine NHS data to monitor readmissions and colectomies, and with an annual questionnaire to capture patient’s quality of life and some healthcare resources. This will provide an opportunity to further update and internally validate the cost-effectiveness model.

In a recent guideline, commissioned by the British Society of Gastroenterology (BSG), for the management of inflammatory bowel disease (IBD) in adults, the committee members recommended that ASUC patients not responding to corticosteroid should be treated with intravenous infliximab or ciclosporin who have not failed a previous thiopurine therapy [48]. However, the use of cicloporin, compared to infliximab, for managing ASUC patients by clinicians appears to be less prevalent in the UK [49]. This is partly because of the healthcare professionals’ preference for infliximab in treating ASUC patients, mainly owing to resource intensive nursing involvements with intravenous ciclosporin [50].

Infliximab is more expensive, but has been widely used due to its simpler administration. Because of the availability of new anti-TNF biosimilars in the market, the cost of infliximab has been falling [25], and the cost still remains higher than ciclosporin. The study findings provide evidence in favour of ciclosporin on the cost-effectiveness decisions for the management of the NHS ASUC patients with about 95% certainty. However, the practical challenge with treatment decisions remains in the balance of clinical equivalence of two alternative treatments and easier dealing with infliximab by clinicians. With cheaper biosimilars available in the market, clinicians tend to use infliximab due to its easier administration and partly due to non-significant difference in health effects between two treatments. Furthermore, although policy makers’ reimbursement decisions are not based on only economic grounds, the authors argue that the opportunity costs to other patients have to be accounted for while choosing a treatment that is not cost-effective. Because, a wrong decision is unlikely to bring benefits to patients and also to lead to an inefficiency in the healthcare system.

## Conclusions

From a long-term cost-effectiveness modelling of data from a pragmatic RCT ciclosporin produced positive incremental net health benefit relative to infliximab. Our results indicated that ciclosporin is dominant compared with infliximab for the treatment of the NHS ASUC patients, however, these results are subject to some degree of uncertainty, mostly due to non-significant difference in health outcomes between competing drugs. In the long-term, ciclosporin was cost-saving relative to infliximab even though it (ciclosporin) was associated with an estimated higher colectomy rate, and remained cost-effective against an NHS WTP threshold value of up to £20,000.

## Supplementary Information

**Additional file 1:** **Appendix 1.** A Decision Tree Model was developed based on events occurred in CONSTRUCT trial patients over 24 months follow up. The decision tree has the same structure (pathways) for both infliximab and ciclosporin groups. ‘Intitial’ period represents 0-3 months, ‘mid-term’ represents 4-12 months and ‘late’ represents 13-24 months of the 2 years duration in the decision tree. **Appendix 2.** Long-term Markov model for patients in remission beyond 24 months. Patients can move across different health states in the Markov model. After surgery patients can achieve surgical remission or experience surgical complications, achieve surgical remission afterwards, or they can die. **Appendix 3.** Cumulative number of colectomies (%) in the trial at various follow-up time points for two treatment groups. **Appendix 4.** Model parameters in the Markov Model. **Appendix 5.** The unit costs of two trial drugs. **Appendix 6.** Kaplan-Meier survival curves showing colectomy-free survival for infliximab versus ciclosporin over the follow-up period in the CONSTRUCT trial. **Appendix 7.** Weibull regression predicted transition probability of colectomy for both infliximab and ciclosporin groups.

## Data Availability

Data that support the findings of this study are kept with the corresponding author (MFA) and can be sought after a reasonable request. Restrictions apply to access to CONSTRUCT trial data which are kept with trial’s LPI (JGW). No additional data are available.
