# Supplementary material for: Infliximab versus ciclosporin in steroid resistant acute severe ulcerative colitis: a model-based cost-utility analysis of data from CONSTRUCT pragmatic trial
Source: BMC Health Serv Res. 2023 Mar 8;23:226. doi: 10.1186/s12913-023-09233-w (PMC9993375; doi:10.1186/s12913-023-09233-w)
Supplement: Supplementary file 1 — Additional file 1: Appendix 1. A Decision Tree Model was developed based on events occurred in CONSTRUCT trial patients over 24 months follow up. The decision tree has the same structure (pathways) for both infliximab and ciclosporin groups. ‘Intitial’ period represents 0-3 months, ‘mid-term’ represents 4-12 months and ‘late’ represents 13-24 months of the 2 years duration in the decision tree. Appendix 2. Long-term Markov model for patients in remission beyond 24 months. Patients can move across different health states in the Markov model. After surgery patients can achieve surgical remission or experience surgical complications, achieve surgical remission afterwards, or they can die. Appendix 3. Cumulative number of colectomies (%) in the trial at various follow-up time points for two treatment groups. Appendix 4. Model parameters in the Markov Model. Appendix 5. The unit costs of two trial drugs. Appendix 6. Kaplan-Meier survival curves showing colectomy-free survival for infliximab versus ciclosporin over the follow-up period in the CONSTRUCT trial. Appendix 7. Weibull regression predicted transition probability of colectomy for both infliximab and ciclosporin groups. [file 12913_2023_9233_MOESM1_ESM.pdf]

Supplementary Documents

Appendix 1

A Decision Tree Model was developed based on events occurred in CONSTRUCT trial patients over 24 months follow up. The decision tree has the same structure (pathways) for both infliximab and ciclosporin groups. 'Intitial' period represents 0-3 months, 'mid-term' represents 4-12 months and 'late' represents 13-24 months of the 2 years duration in the decision tree.

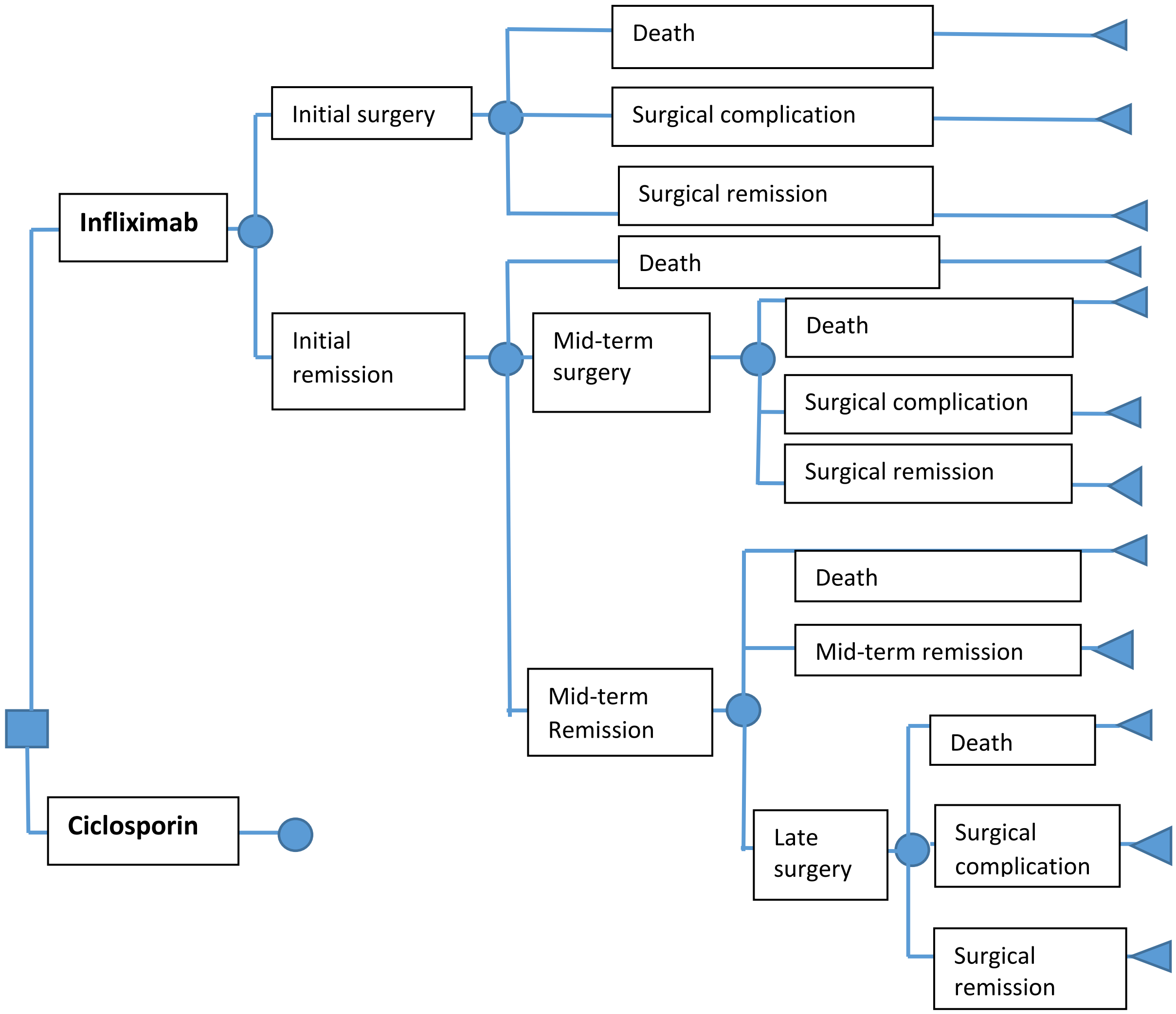

Appendix 2

Long-term Markov model for patients in remission beyond 24 months. Patients can move across different health states in the Markov model. After surgery patients can achieve surgical remission or experience surgical complications, achieve surgical remission afterwards, or they can die.

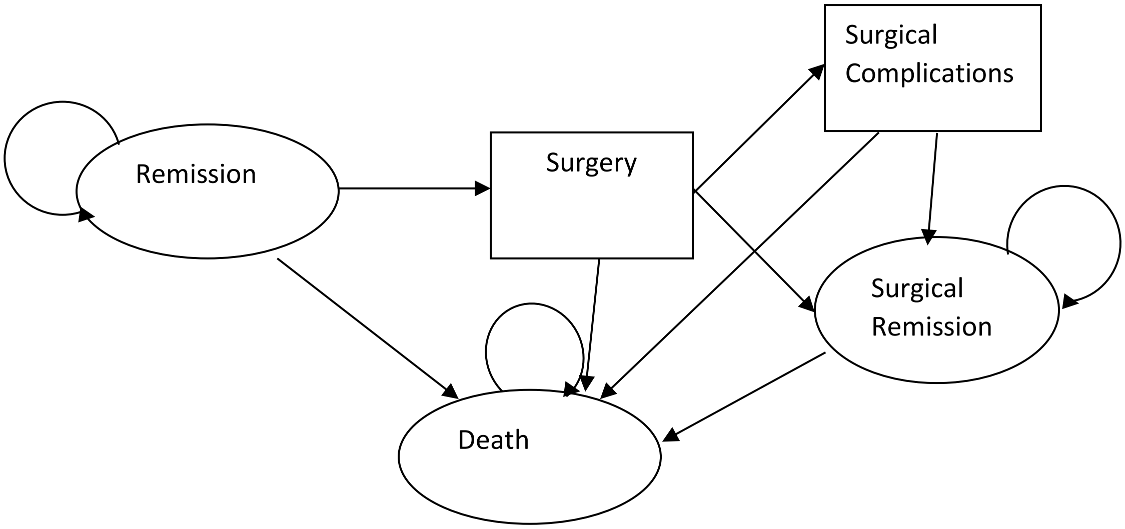

Appendix 3

Cumulative number of colectomies (%) in the trial at various follow-up time points for two treatment groups

Follow-up	Ciclosporin (n=135)		Infliximab (n=135)	
	Number	%	Number	%
3 months FU	42	31.1	38	28.1
12 months FU	62	45.9	47	34.8
24 months FU	65	48.1	54	40.0
30 months FU	65	48.1	55	40.7

Appendix 4

Model parameters in the Markov Model

	Base value	Standard error	Source
Inflx_ lambda	0.1113	0.0967	Weibull regression; CONSTRUCT trial
Inlx_gamma	0.3927	0.0472	Weibull regression; CONSTRUCT trial
Ciclos_lambda	0.1864	0.144	Weibull regression; CONSTRUCT trial
Ciclos_gamma	0.4497	0.0474	Weibull regression; CONSTRUCT trial
Yearly mortality rate from remission	0.048		Roberts et al. (2007)
Yearly mortality rate after surgery	0.046		Roberts et al. (2007)
Utility Values			
Remission_Inflx	0.852	0.04	CONSTRUCT trial
Remission_Ciclos	0.923	0.019	CONSTRUCT trial
Utility_Surgery_Inflx	0.761	0.06	CONSTRUCT trial
Utility_Surgery_Ciclos	0.821	0.06	CONSTRUCT trial
Post-surgery remission	0.692	0.07	CONSTRUCT trial

Note: Yearly mortality rate based on Roberts et al. (2007) [30]

Appendix 5

The unit costs of two trial drugs

**Drug**	**Dose**	**Unit cost (£)**
Ciclosporin (Sandimmun)	50mg/ml, 1-ml ampoules	£1.94
Oral ciclosporin	100 mg, 30-capsule pack	
Neoral		£72.57
Deximune (Dexcel Pharma Ltd.)		£51.30
Capimune (Mylan)		£51.30
Infliximab (Remicade)	100-mg vial	£419.62

Source: British National Formulary (BNF, 2015)

Appendix 6

Kaplan-Meier survival curves showing colectomy-free survival for infliximab versus ciclosporin over the follow-up period in the CONSTRUCT trial

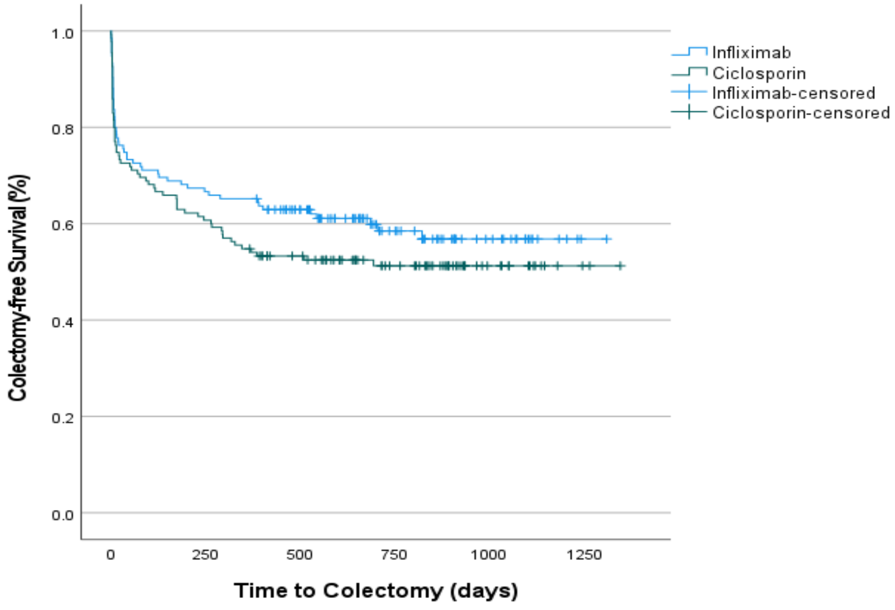

Appendix 7

Weibull regression predicted transition probability of colectomy for both infliximab and ciclosporin groups

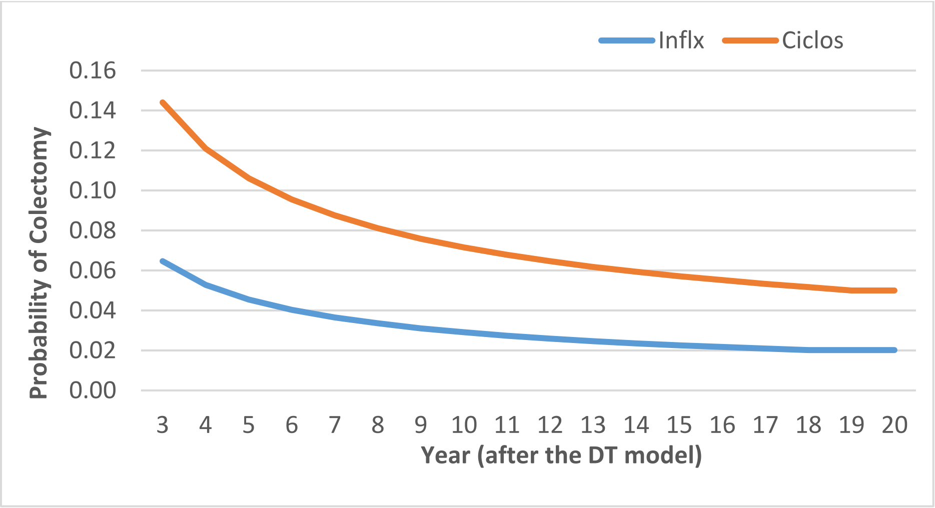

Reference

Joint Formulary Committee. British National Formulary (Online). London: BMJ Group and Pharmaceutical Press. URL: www.medicinescomplete.com (accessed 28 June 2015)
